# Supplementary material for: Investigating the influence of physiologically relevant hydrostatic pressure on CHO cell batch culture
Source: Sci Rep. 2021 Jan 8;11:162. doi: 10.1038/s41598-020-80576-8 (PMC7794228; doi:10.1038/s41598-020-80576-8)
Supplement: Supplementary file 1 — Supplementary Information 1. [file 41598_2020_80576_MOESM1_ESM.zip › S1/IgG/Jan17 standard curve/2/0.pdf]

Data File C:\Users\P...ts\ChemStation\1\Data\TK_CHO_IgG 2019-01-17 15-00-19\005-1-0ug_ml.D
Sample Name: 0ug/ml

===

Acq. Operator : SYSTEM   Seq. Line : 5
Sample Operator : SYSTEM
Acq. Instrument : HPLC-0039   Location : 1
Injection Date : 1/17/2019 3:18:27 PM   Inj : 1
Inj Volume : 100.000 µl
Sequence File : C:\Users\Public\Documents\ChemStation\1\Data\TK_CHO_IgG 2019-01-17 15-00-19\TK_CHO_IgG.S
Method : C:\Users\Public\Documents\ChemStation\1\Data\TK_CHO_IgG 2019-01-17 15-00-19\CHO_IgG_LC.M (Sequence Method)
Last changed : 12/4/2018 1:38:41 PM by SYSTEM

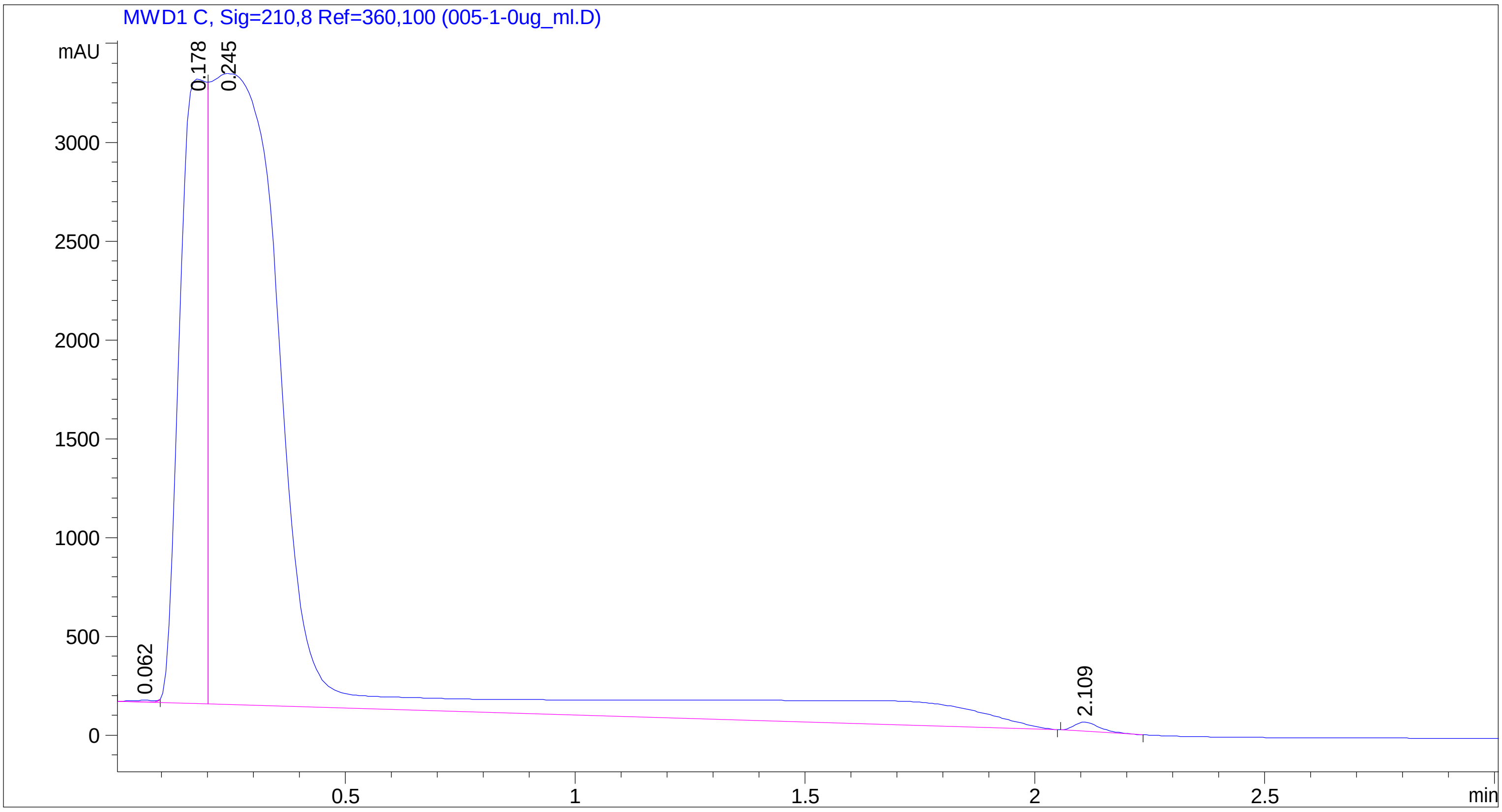

===

Area Percent Report

===

Sorted By : Signal
Multiplier : 1.0000
Dilution : 1.0000
Use Multiplier & Dilution Factor with ISTDs

Signal 1: MWD1 C, Sig=210,8 Ref=360,100

Peak #	RetTime [min]	Type	Width [min]	Area [mAU*s]	Height [mAU]	Area %
1	0.062	BV E	0.0495	25.90924	8.21742	0.0496
2	0.178	VV R	0.0626	1.26365e4	3160.31689	24.2051
3	0.245	VB	0.1858	3.93631e4	3192.74438	75.3999
4	2.109	BBA	0.0591	180.33104	46.65652	0.3454

Totals : 5.22058e4 6407.93522

===

*** End of Report ***
